# Cells containing aragonite crystals mediate responses to gravity in *Trichoplax adhaerens* (Placozoa), an animal lacking neurons and synapses

**DOI:** 10.1371/journal.pone.0190905

**Published:** 2018-01-17

**Authors:** Tatiana D. Mayorova, Carolyn L. Smith, Katherine Hammar, Christine A. Winters, Natalia B. Pivovarova, Maria A. Aronova, Richard D. Leapman, Thomas S. Reese

**Affiliations:** 1 Laboratory of Neurobiology, National Institute of Neurological Disorders and Stroke, National Institutes of Health, Bethesda, Maryland, United States of America; 2 Laboratory of Developmental Neurobiology, Koltzov Institute of Developmental Biology, Russian Academy of Science, Moscow, Russia; 3 Central Microscopy Facility, Marine Biological Laboratory, Woods Hole, MA, United States of America; 4 Laboratory of Cellular Imaging and Macromolecular Biophysics, National Institute of Biomedical Imaging and Bioengineering, National Institutes of Health, Rockville Pike, Bethesda, Maryland, United States of America; University of California Irvine, UNITED STATES

## Abstract

*Trichoplax adhaerens* has only six cell types. The function as well as the structure of crystal cells, the least numerous cell type, presented an enigma. Crystal cells are arrayed around the perimeter of the animal and each contains a birefringent crystal. Crystal cells resemble lithocytes in other animals so we looked for evidence they are gravity sensors. Confocal microscopy showed that their cup-shaped nuclei are oriented toward the edge of the animal, and that the crystal shifts downward under the influence of gravity. Some animals spontaneously lack crystal cells and these animals behaved differently upon being tilted vertically than animals with a typical number of crystal cells. EM revealed crystal cell contacts with fiber cells and epithelial cells but these contacts lacked features of synapses. EM spectroscopic analyses showed that crystals consist of the aragonite form of calcium carbonate. We thus provide behavioral evidence that *Trichoplax* are able to sense gravity, and that crystal cells are likely to be their gravity receptors. Moreover, because placozoans are thought to have evolved during Ediacaran or Cryogenian eras associated with aragonite seas, and their crystals are made of aragonite, they may have acquired gravity sensors during this early era.

## Introduction

*Trichoplax adhaerens*, a member of the ancient phylum Placozoa, inhabits warm oceans where it adheres to and feeds upon biofilms [1,2]. Information about its feeding behavior is derived from observing a clone maintained in the laboratory for over 40 years, where it grazes algae on the bottom of the dish or at the air-water interface [2,3]. *Trichoplax* has only six cell types, 85% of which are concentrated in a thick epithelium that covers its ventral surface, the lower surface when the animal is on the bottom of the dish and the upper surface when it is at the air-water interface [4]. Animals glide on the substrate, powered by ciliated cells in the ventral epithelium [2–6]. When a gliding animal encounters algae, gland cells distributed around the edge of the animal secrete a peptide that arrests ciliary beating, causing the animal to cease gliding. Then, lipophil cells, which are distributed throughout the ventral epithelium, release enzymes externally that lyse the algae, and the lysate is endocytosed by the ventral epithelial cells [7]. Fiber cells are not represented on the surface of the animal, but lie in a space between the ventral and thin dorsal epithelia, where they give rise to long branching processes that contact other cells [4,6,8]. Crystal cells, the least prevalent type, also reside in the space between the ventral and dorsal epithelia but, unlike fiber cells, occur only in a narrow band ~20 μm from edge of the animal. A birefringent crystal is centered in its cell body in a cup formed by the extremely flattened nucleus. The crystal is surrounded by mitochondria but the cytoplasm in the rest of the crystal cell is remarkably clear of organelles [4,9]. Could this crystal be a statolith?

Statocysts in animals may be ciliated or non-ciliated [10,11]. While ciliated statocysts are typical through a wide range of animals, non-ciliated statocysts are apparently more ancient, occurring as well in Plants and Protozoa. Indeed, both non-vascular plants and Embryophytes owe root positive gravitaxis to minute intercellular amyloplasts or statoliths in root cells [12–14]. Some protists also determine the direction of gravity by means of statoliths in their cytoplasm [15–18]. A few species of hydroids [19], free-living flat worms [20–24] and ribbon worms [25], ecdysozoans [11], and ascidian larvae [26] deploy non-ciliated statocysts and if crystal cells of placozoans are statocytes, they belong to the non-ciliated type. In order to determine whether crystal cells in *Trichoplax* are gravity sensors, we used serial section EM to analyze the internal structure of crystal cells as well as their relationships to surrounding cells, and then determined the responses of the crystals and the behavior of *Trichoplax* to changes in the direction of gravity. We also used spectrographic analyses to determine the composition of the crystal and conclude that the crystal cells in *Trichoplax* may have evolved in or before the Ediacaran era.

## Materials and methods

### Materials

*Trichoplax adhaerens* of the Grell (1971) strain, a gift from Leo Buss (Yale University), were maintained in artificial seawater (ASW) (Instant Ocean), specific gravity 1.024, at room temperature with 1% Micro Algae Grow (Florida Aqua Farms). Trichoplax medium was changed once a week. *Trichoplax* were fed red algae (*Rhodamonas salina*, Provasoli-Guillard National Center for Culture of Marine Plankton).

### Electron microscopy

*Trichoplax* were high pressure frozen and then freeze substituted and embedded as described previously [4]. For transmission electron microscopy, 70 nm sections were collected on formvar, carbon coated single slot grids (Electron Microscopy Sciences, USA). They were examined at 120–200 KV in a JEOL JEM 200-CX electron microscope (Japan) and photographed with an AMT camera mounted below the column.

For scanning electron microscopy, sample blocks were cut on the PowerTomeXL ultramicrotome consisting of a tape collection unit the “ATUM”, RMC Boeckler Instruments, Inc. (USA). Sections 70 nm thick were collected onto glow discharged Kapton tape (EMS) and the tape was cut to size to mount on a mechanical grade silicon wafer (University Wafer, USA) using double sided carbon tape (EMS). The wafer was carbon coated using graphite rods (EMS) in a Denton DV-502 Vacuum Evaporator (Denton Vacuum, USA). SEM images were captured in backscatter mode using a Zeiss SUPRA 40VP Gemini Scanning Electron Microscope and the Atlas5 software with Zeiss SMARTSEM. Images of serial sections through two crystal cells collected in backscatter mode were uploaded into Fiji 2.0.0 (USA), where they were aligned and segmented. Rendering was performed in Amira 5.4.4 (Germany).

### Fluorescent labelling

Silane covered glass cover slips or Lab-Tek II coverslip chambers (Thermo Fisher Scientific) were used make samples for laser scanning confocal microscopy. Silanised cover slips were made by soaking them in nitric acid, incubating them in (3-Aminopropyl)triethoxysilane (Sigma-Aldrich, China), and rinsing them in acetone before drying.

To visualize nuclei, *Trichoplax* attached to silanised cover slips were fixed in a mixture of paraformaldehyde (4%, EMS, USA) and Hoechst dye (1:2000; Life Technologies, USA) in buffered ASW (NaCl 400 mM; MgCl_2_ 5 mM; CaCl_2_ 2mM; sucrose 300mM; HEPES 30mM; pH 7.4) for two hours. Samples were then rinsed thrice with PBS and mounted in Vectashield (Vector Laboratories, USA) on glass slides with EM grids sandwiched between as spacers. Images were collected with a 63x numerical aperture (NA) 1.4 objective and 405 nm illumination and on a LSM 510 confocal microscope (Carl Zeiss Microscopy, Thornwood, NY) or a SP8 confocal microscope (Leica Microsystems, Inc., Exton, PA) with Huygens deconvolution software (Scientific Volume Imaging, Hilversum, Netherlands).

To visualize actin filaments, animals first were incubated in Hoechst dye (diluted 1:1000 in ASW) for 15 minutes, and then simultaneously fixed and stained in a solution consisting of 4% paraformaldehyde in PBS with 0.5 M NaCl, 0.2% saponin, and Oregon Green Phalloidin (12.5:1000) for 1 hour. Images were captured with a 63X NA 1.4 objective and 405 and 488 nm illumination on a LSM 880 with an Airyscan detector (Carl Zeiss Microscopy).

### Crystal dynamics in tilted *Trichoplax*

*Trichoplax* were deposited on silanised cover slips on the bottom of a Petri dish in ASW. After animals attached to the cover slips, some coverslips were tipped up to 90° by placing them in a 50 ml tube half filled with ASW, and the down side of each tipped cover slip was marked. Controls were left undisturbed in their Petri dishes. Cover slips tilted up for either 2 or 30 min were gently transferred without changing their orientation into another 50 ml tube half filled with PFA/Hoechst mixture (see above). The ASW on horizontal cover slips was also replaced with the staining mixture. Samples were then processed as described above and examined under the LSM 510 microscope. All crystal cells found in unfolded areas in each *Trichoplax* were photographed as stacks of optical sections at 1 μm intervals. Collected images were then analyzed using Fiji or LSM 510 4.0 SP1 software.

The position of each crystal was assessed choosing the images with the sharpest focus of the crystal cell nucleus, outer membrane, and the crystal itself, and its position scored as up, down or center. For tilted animals, the image plane was parallel to the gravity vector, so up and down directions corresponded to the direction of the gravity vector. The datasets were randomized and examined blind by an experienced microscopist to record the positions of every crystal within the cell body. Only those *Trichoplax* were counted that had more than ten crystal cells in which crystal positions could be determined. Also, excluded from measurements were what appeared to be immature crystal cells with oval nuclei and tiny (less than 1 μm) crystals. Results were expressed as the percent of crystal cells in each animal manifesting up, down or center crystal positions. The means of the percentage of crystal cells with a crystal in up, or down, or center partitions for each group were used to create a graph. In total, crystal positions were measured in six untilted, control, animals (133 crystal cells); in two animals tilted for two minutes (97 crystal cells), and in seven animals tilted for 30 minutes (178 crystal cells). Error bars are standard errors of mean (SEM). Statistical analyses utilized t-tests (PAST 3.14 Software, University of Oslo, Norway). Comparison of the percentage of crystal cells with up and down positions of crystals in not-tilted animals showed a significant difference from tilted animals (p < 0.05).

### Crystal extraction

*Trichoplax* were briefly rinsed in deionized water and then quickly transferred into 200 μm of fresh deionized water or deionized water supplemented with 1% sodium dodecyl sulfate (SDS) which dissolved the animal, leaving no visible debris. A small drop (~2 μm) of this solution was then transferred to formvar coated 200 μm mesh copper grids (EMS, USA). Drops were followed with a light microscope equipped with DIC optics while they dried on the grid, thereby tracing all the birefringent crystals caught in the drop. The positions of all crystals were mapped after the grid dried. Grids with extracted crystals were then imaged in a JEOL JEM 200-CX electron microscope. Crystals extracted with deionized water were also subjected to electron microscopic spectral analyses (see below).

### X-ray spectroscopy

Crystals were imaged and analyzed in an analytical electron microscope Zeiss EM912 (Carl Zeiss Microscopy, Thornwood, NY). X-ray spectra were recorded using a Linksystem Pentafet EDX detector (Oxford Instruments, Concord, MA) and processed by established procedures [27].

### Electron energy loss spectroscopy (EELS)

Distributions of carbonate C = O bonds and elemental calcium were mapped using electron energy loss spectroscopic imaging (EELSI) in a transmission electron microscope (TEM) operating in the scanning TEM (STEM) mode. EELSI data cubes were recorded at an accelerating voltage of 300 kV by means of Tecnai TF30 electron microscope (FEI, Inc.), equipped with a Quantum Imaging Filter (Gatan Inc.), which was controlled using the Gatan Digital Micrograph software package. A high-angle annular dark-field (HAADF) detector (Fischione Instruments, Inc.) was used to acquire STEM images containing 2048 x 2048 pixels. Small sub-regions of the STEM images were selected and EELSI data cubes were acquired with approximately 30 pixels x 30 pixels x 2048 channels and an energy loss range of 227 eV to 739 eV and 0.25 eV/channel. The pixel size was 41 nm x 41 nm and spectral dwell time per pixel was 0.02 second.

To extract the signal at the C K-edge originating specifically from the carbonate C = O bonds, three energy windows of width 2.5 eV were selected after the spectral background was fitted to an inverse power law and subtracted. The first was centered on the 1s –> π* peak at an energy loss of 289.25 eV and two background windows, one below the 1s –> π* peak at an energy loss of 286.0 eV, and another above the 1s –> π* peak at an energy loss of 291.5 eV. The signal $S_{1s\to \pi^{*}(C=O)}$ can then be approximated by:

$$S_{1s\to \pi^{*}(C=O)}=S_{eV-290.0\;eV}-\frac{S_{284.8eV-287.3\;eV}+S_{290.3\;eV-292.8\;eV}}{2}$$

To obtain the integrated core-edge signals for the Ca L_2,3_ edge and the O K-edge, the spectral background was fitted to an inverse power law in the pre-edge window from 345 eV– 355 eV for calcium and into the pre-edge window from 530 eV– 550 eV for oxygen. The extrapolated background was then subtracted to give the net core edge signals. To check whether crystals have magnesium, we looked for a Mg K edge at 1306 eV in the EEL spectrum.

### Behavior

The behavior of animals on vertical substrates was compared with that of animals on horizontal substrates to test reactions to changes in direction of gravity. Experiments were carried out in a darkened room at a constant temperature in light coming from a precision light box of adjustable intensity and heat control (Northern Light Technologies, Inc., Canada). Time-lapse recordings used a Canon EOS 5D Mark III 22.3 MP Digital Camera equipped with Canon MP-E 65mm f/2.8 1-5x Macro Photo lens. EOS Utility 2.13.40.2 software was used to control the camera and collect images.

Before an experiment, *Trichoplax* were washed two times with ASW to remove *Trichoplax* medium and algae. Then the animals were examined for the quantity of crystals with differential-interferential contrast optics. Animals were divided into two groups: those with plentiful crystals spread throughout the whole perimeter, and those with sparse crystals (0–8). Since the latter are very rare in our cultures, hundreds of *Trichoplax* were culled to collect thirty-three crystal depleted animals. Animals with normal or sparse crystals were transferred to a glass slide in a glass container (Wheaton, USA) filled with ASW, rested for 10 min. The glass container was positioned on a plastic Petri dish which was centered on the lighting surface of the light box (illuminance 276 lux). The slide was then tilted vertically (~90°) and fixed in the vertical position using the ribs on the sides of a container. A camera was positioned on the same plane as the reservoir containing animals to record images from the side. Images were taken every 30 seconds during two hours during which there was no measurable heating. Fiji software was used to trace tracks of individual animals. To measure net up/down displacement, we placed a dot in the center of each animal on each image and recorded their positions manually. Each value was then subtracted from the first one to derive net displacement. Only animals which did not contact other animals during the course of experiment were measured. In total, we measured positions of 49 *Trichoplax* with plentiful of crystals and 33 *Trichoplax* with sparse crystals. Results are expressed as the mean displacement from the initial point plotted every 30 seconds. Error bars are SEM.

## Results

### Electron microscopy of crystal cells

**Fig 1:**
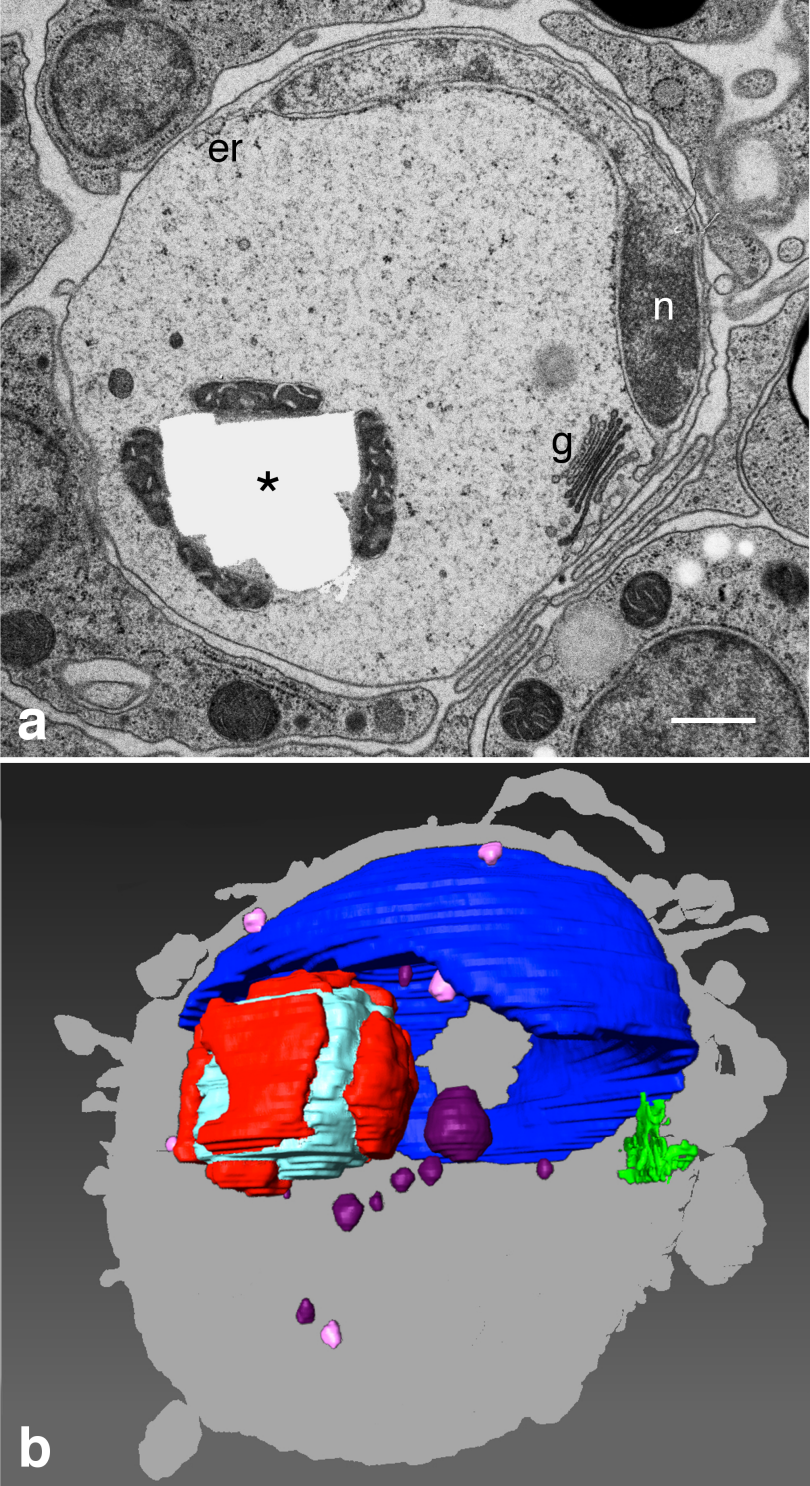
Crystal cells typically contain a single crystal. (a) Section of crystal cell by TEM shows void previously occupied by crystal (asterisk). Crystal void is surrounded by mitochondria. Flattened nucleus of crystal cell (*n*) is applied to the cell membrane. Sparse additional inclusions include light and dark vesicles and a Golgi apparatus (*g*). (b) Reconstruction of interior of crystal cell from 123 serial sections. Mitochondria (red) surround crystal (light blue). The crystal complex sits in the cup formed by the nucleus (blue), and a Golgi apparatus (green) flanks the nucleus. Light and dark vesicles in light and dark pink. Crystal cell silhouette is shown in gray. Abbreviations: *g*–Golgi apparatus; *er*–endoplasmic reticulum; *n*–nucleus. Scale bar 500 nm.

Crystal cells distributed near the upper edge of the disk-shaped body of *Trichoplax* contain birefringent intracellular crystals [4]. We analyzed crystal cells by serial section SEM to understand the three-dimensional arrangement of the intracellular crystal with respect to other organelles. Crystals in animals embedded in epoxy resins typically disappeared during sectioning, leaving a void corresponding in shape to the crystal. In some instances, fragments in the shape of a broken crystal persisted (S1 Fig). Judging from these voids, crystals typically were rhomboid or square-shaped, and measured 1.5–2.5 μm per side (Fig 1A and 1B; S1 Fig), although some were ovoid or lens-shaped. Each crystal was surrounded and enclosed by 5–6 mitochondria, which were in close contact with it. There were no mitochondria elsewhere in the cell.

Crystal cells had a cup-shaped nucleus, some manifesting perforations in their wall. (Fig 1A and 1B; S1 Movie). The nuclei were tightly apposed to one side of the cell, covering up to a half of the internal surface of the plasma membrane. The outer membrane of the nuclear envelope was continuous with the endoplasmic reticulum deployed around the rim of the cup (Fig 1A; S2 Fig; S2 Movie) and a Golgi complex typically lay near the rim of the nuclear cup (Fig 1A and 1B). A few separate vesicles of different size and electron density were scattered in the cytoplasm (Fig 1A and 1B). Generally, the cytoplasm of a crystal cell had strikingly fewer inclusions and organelles compared to other types of cells (Fig 1A).

### Distribution and orientation of crystal cells

The numbers and distributions of crystal cells were inferred from the locations of birefringent crystals observed with polarized light in living animals <200 μm to over 1.5 mm in diameter. The numbers of crystals varied between animals [4,9], ranging from zero to 150. However, animals lacking crystals were rare; most animals had multiple crystals spaced approximately 20–30 μm apart around their entire circumference. Most crystals cells were located within ~ 20 μm of the edge of the animal, although a few were further toward the interior. Crystals measured in transmitted light varied in size from 1 to 3 μm, consistent with the EM observations. Some crystal cells with a very small crystal lacked a cup-shaped nucleus, and are presumed to be immature cells (not shown).

**Fig 2:**
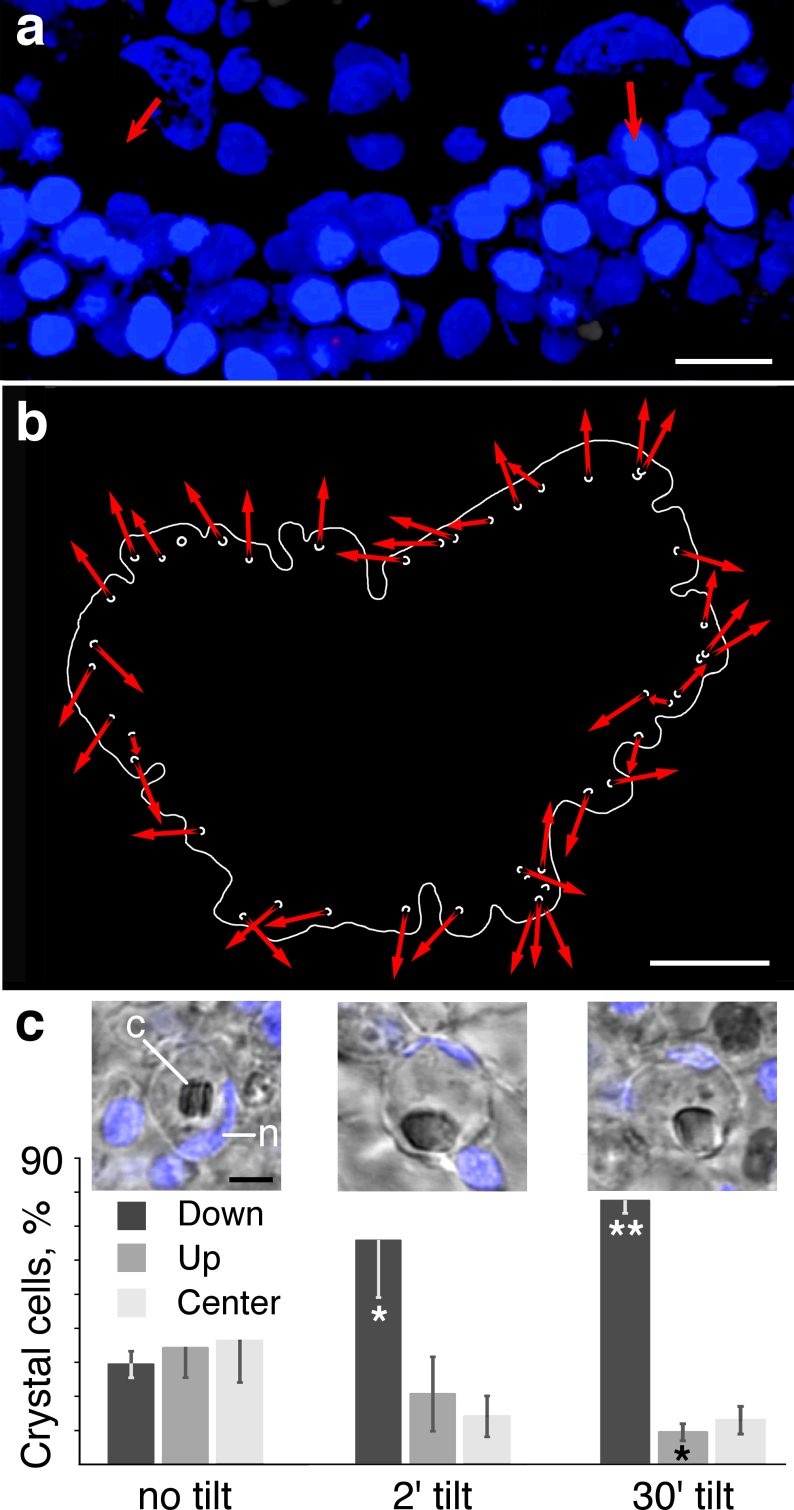
Orientation of crystal cells and position of crystal. (a) Cup shaped nuclei of two crystal cells are apparent in an *en face* view of the edge of an animal stained with Hoechst nuclear dye. Orientations of the cups are indicated by red arrows. Oval-shaped nuclei of epithelial cells are tightly packed near the edge of the animal (below). Volume rendering of a series of confocal optical sections was created with Leica 3D imaging software. (b) Map of the positions and orientations of the cup-shaped nuclei of crystal cells. Outline traces the edge of the animal. Orientations of the openings of the cups are indicated by red arrows in mouths of the cups. Most cup openings are oriented towards the perimeter. (c) Effect of gravity on the positions of crystals inside crystal cells. Insets show the positions of crystals (*c*) inside their crystal cell in an animal on a horizontal surface (left), or tilted vertically for 2 min (center) or 30 min (right). Graphs compares the proportion of crystal cells with crystal shifted *up*, *down* or in the *center* under the three conditions. Error bars are SEM. *—p<0.05. **—p<0.005. Abbreviations: *c*–crystal; *n*–crystal cell nucleus. Scale bars: a– 5 μm; b– 100 μm; c– 2 μm (insets).

The orientations of crystal cells were assessed by a combination of fluorescence and transmitted light microscopy on fixed animals stained with Hoechst nuclear dye. The fluorescence images revealed the orientations of the cup-shaped nuclei (Fig 2A and 2B) while the transmitted light images revealed the outlines of crystals as well as those of the crystal cell containing them (Fig 2C, insets). The cup shaped nuclei consistently manifested preferred orientations, with the opening of the cup partly enclosing the crystal and facing the perimeter of the animal (Fig 2A), so the mouth of almost every cup was directed toward the outer edge of the animal (Fig 2B)

### Effect of direction of gravity on position of crystals

How positions of crystals within crystal cells change under the influence of gravity was determined by comparing animals maintained on horizontal surfaces (control) with animals on surfaces tipped up to vertical for two or 30 minutes (tipped). The positions of 408 crystals were monitored in 15 *Trichoplax*, where every crystal in clear view was scored as *up*, *down* or *center*. Animals with ten or fewer crystals were not sampled. The crystals in 133 crystal cells in six control animals were essentially equally distributed among the *up* (34% ± 21; mean±SD; Fig 2C) and *down* (29% ± 10) partitions. In contrast, crystals shifted downwards in tipped animals. In two animals tipped for two minutes, 65% ± 24 (mean±SD, Fig 2C) of 97 crystal cells had a crystal in the *down* position while 21% ± 15 of cells had a crystal in the *up* direction. The percent of crystals in *down* position was significantly greater compared with the percent of crystals in *down* position in control animals (p < 0.05). In seven *Trichoplax* with total of 178 crystal cells tilted for 30 min, more crystals occurred in the *down* (78% ± 10) and fewer were found in the *up* partition (9% ± 7, Fig 2C). The result after the 30 min tilt differed significantly from the control crystals for both up (p < 0.05) and down (p < 0.005) partitions. While the positions of the crystals within crystal cells appeared to shift under the influence of gravity, the orientation of the cup-shaped nuclei remained facing the outer edge of the animal under both conditions.

### Interactions of crystal cells with other cells

**Fig 3:**
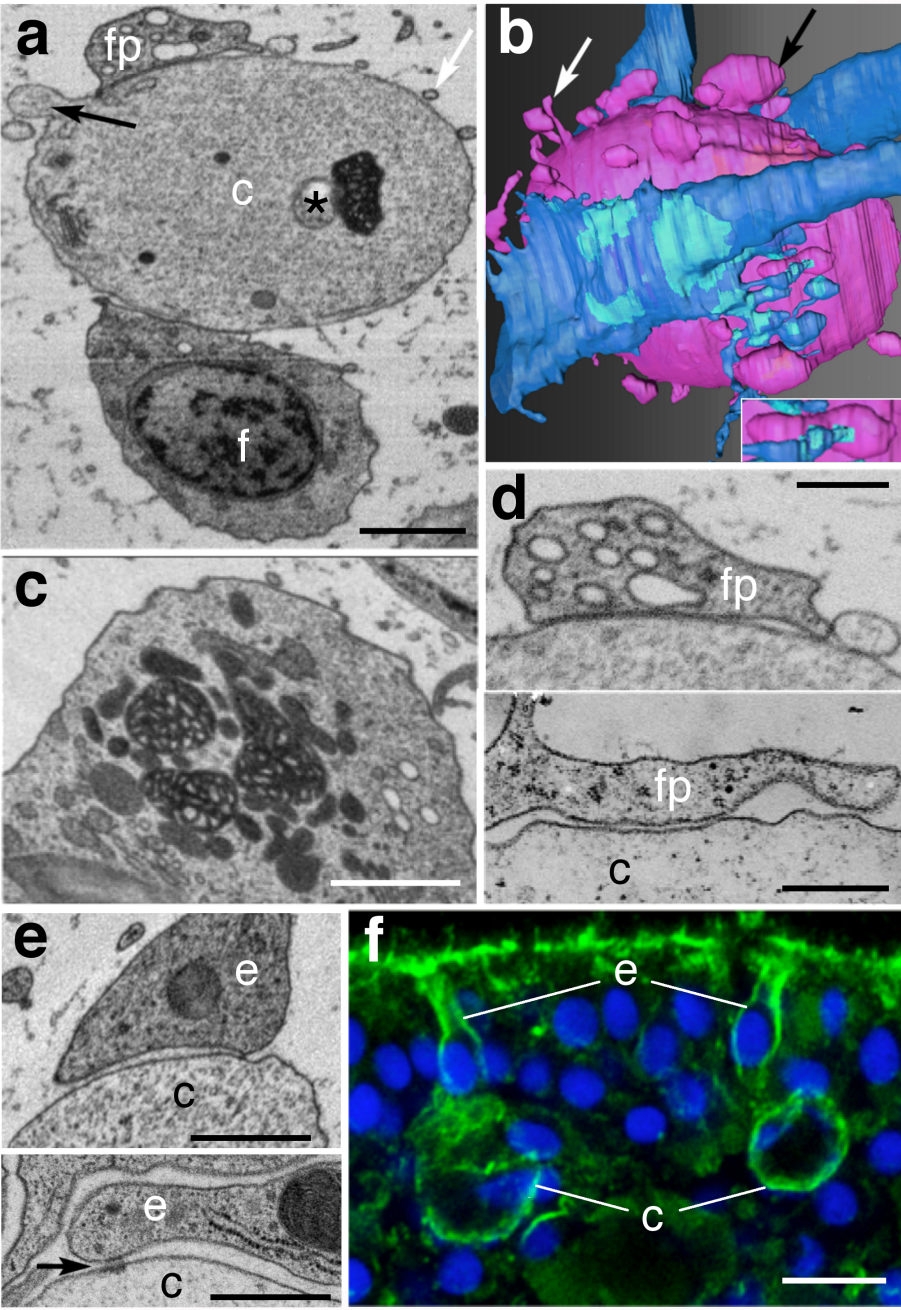
Crystal cells are contacted by fiber and epithelial cells. (a) Crystal cell (*c*) is contacted by fiber cell process (*fp*, above) and the cell body of a fiber cell (*f*, below). The identities and relationships of the cells were established by reconstructing 123 serial sections acquired with backscatter SEM. Asterisk marks the tiny void at the tip of the missing crystal. Crystal cell gives rise to thin processes (white arrows) and as well as larger processes (black arrows). (b) Surface rendering derived from the reconstruction shows that the crystal cell (magenta) is contacted by two fiber cells and their processes (blue). Cyan areas outline areas of contact of the fiber cell with the crystal cell. Crystal cells have numerous thick and thin side branches (black and white arrows, respectively). Inset enlarges a fiber cell process contacting a side branch of the crystal cell. (c) Section from the same stack shows a fiber cell body lying under the crystal cell contains a cluster of dark mitochondria and paler organelles, forming the mitochondrial complex that is a hallmark of fiber cells. (d) Crystal cell (*c*) in thin section SEM (above) is in tight contact with fiber cell process (*fp*). Comparable fiber cell contact in TEM thin section (below), where no junctional structures are apparent along the contact zone. (e) Appositions between a crystal cells (*c*) and epithelial cells (*e*) in thin section SEM (top) and TEM (below). Arrow indicates filamentous material spanning the cleft between the cells. (f) Edge of animal stained with Hoechst (blue) and Phalloidin (green) viewed by confocal microscopy. Maximum intensity projection of three sequential 0.185 μm optical sections. Phalloidin staining shows abundant actin filaments lining the plasma membranes of two crystal cell (*c*) and in the epithelial cells (*e*) that contact each of them. Abbreviations: *c*–crystal cell; *e*–epithelial cell; *f*–fiber cell; *fp*–fiber cell process. Scale bars: a, c– 1 μm; d– 200 nm; e– 500 nm; f– 5 μm.

The typical positioning of crystal cells ~ 20 μm from perimeter of the animal as well as their fixed orientation might depend on their being anchored by interactions with other cells. Serial section electron microscopy showed that crystal cells were regularly contacted by processes of fiber cells (Fig 3A and 3B). The processes varied in contour and diameter and contained numerous electron-lucent vesicles of different sizes up to 600 nm, although none clustered at sites of contact with crystal cells (Fig 3A and 3D). At sites of apposition, the membranes of the two cells were sufficiently close and parallel to suggest that their membranes were attached, although no specialized junctions resembling chemical synapses or gap junctions were apparent (Fig 3D). Some, but not all crystal cells also had extensive contacts with fiber cell bodies. The crystal cells themselves gave rise to a variety of small processes, some very thin (~75 nm, Fig 3A and 3B), others thicker, with narrow necks and enlarged heads up to 1.1 μm in diameter (Fig 3A and 3B, S3 Movie). Some crystal cell processes contacted fiber cell processes (Fig 3B, inset). Crystal cells had a cluster of small vesicles in the vicinity of the Golgi complex and a few larger vesicles elsewhere in their cell bodies but their processes lacked vesicles (Fig 1A; Fig 3A).

In addition to fiber cells, each crystal cell was contacted by one or more epithelial cells (Fig 3E; S3 Movie). The cleft between the crystal cell and epithelial cell for the most part was 40 to 80 nm wide and appeared empty but occasionally included a small adherens type of junction where the cleft narrowed to ~30 nm and was crossed by filamentous material (Fig 3E). Confocal microscopy of animals stained with fluorescent phalloidin to highlight actin filaments revealed that the epithelial cells that contacted crystal cells belonged to the epithelium at the circumference of the animal and were columnar in shape with a narrow ending at the surface, typical of ventral rather than dorsal epithelial cells. Moreover, they differed from other epithelial cells in that they contained prominent bundles of actin filaments around their entire perimeters (Fig 3F). A prominent actin layer also surrounded the cell bodies of crystal cells (Fig 3F).

### Composition of crystals

**Fig 4:**
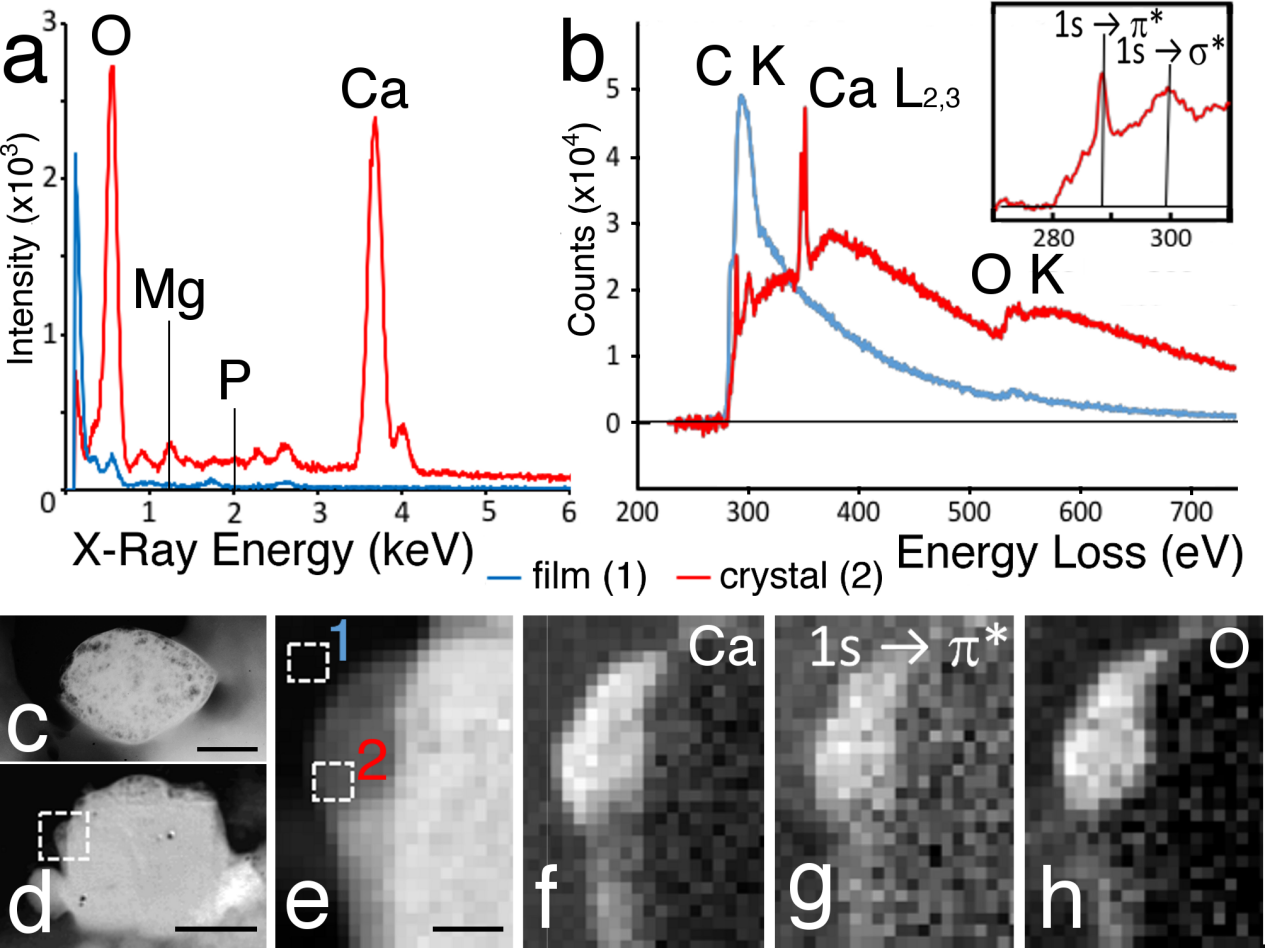
**Chemical composition and appearance of isolated crystals selected in TEM to be analyzed by X-ray (a) and by electron energy loss spectroscopy (EELS) (b).** The x-ray spectrum (a) from a small crystal (c, by TEM, contrast reversed) shows a large calcium peak (red). Blue record corresponds to signal from the support film. EELS data were obtained from the area along the edge of the crystal (box in d, visualized by STEM (HAADF) image), shown enlarged in (e). Boxes in (e) represent two subzones, producing a spectrum from the crystal (Box 2) shown in red in (b) and another from Box 1, over the substrate, shown in blue in (b). Box 2 yields a spectrum characteristic of calcium carbonate (b, red record); the spectrum here shows C K, Ca L_2,3_, and O K edges, together with C K energy loss containing 1s → π* peak at 289.3 eV an (inset) 1s → σ* peak at 300.0 eV. Box 1, control, is located on support film giving spectrum characteristic of amorphous carbon (b, blue line). The whole EELS dataset from boxed region in (d) was used to generate calcium map from Ca L_2,3_ signal (f), carbonate (C = O) map from 1s → π* signal (g), and oxygen map from O K-edge signal (h). Scale bars: c– 600 nm; d– 1 μm; e– 100 nm.

Because crystals appear to be heavy enough to change position inside a crystal cell in response to gravity and break up during sectioning, we assumed that they are in fact little stones. Crystals persisted upon treatment of an animal with 1% sodium dodecyl sulfate to free the crystals from the animal, confirming that crystals are largely inorganic. Extracted crystals viewed under transmission and scanning electron microscopes (Fig 4C and 4D), as predicted from shapes of holes in sections, were lens-shaped, rhomboid or square depending on the angle of view.

Extracted crystals were analyzed in the electron microscope by spectroscopy. X-ray analysis of a whole crystal yielded a large calcium peak (Fig 4A) as well as an oxygen peak. EELS analysis directed at the thin edges of crystals confirmed the presence of abundant calcium and also provided details on the carbon content of a crystal (Fig 4B). Crystals were too thick to obtain useful EELS data except from thin regions close to crystal surfaces (Fig 4C–4G). Spectra from these thin regions showed energy loss near-edge fine structure (ELNES) at the carbon K-edge that was characteristic of carbonate C = O bonds [28,29] with a strong 1s –> π* peak at 289 eV and a 1s –> σ* peak at 300 eV (red spectrum in Fig 4B; 4B inset). The carbon K-edge ELNES from the crystal could easily be distinguished from the ELNES from the nearby support carbon film (blue spectrum in Fig 4B), which did not show any strong core-edge peaks and only a weak feature at 285 eV. The EELSI (Electron Energy Loss Spectroscopic Imaging) data also showed a strong double-peaked feature corresponding to the calcium L_2,3_ edge at 348 eV with spin-orbit splitting of 3.5 eV between the L_3_ and L_2_ peaks, as well as a strong oxygen K at 532 eV (Fig 4B). We did not find any traces of phosphorus and magnesium using either analytic technique. It appeared based on EELS data that the crystals in crystal cells consist of aragonite calcium carbonate.

### Reaction to change in direction of gravity

**Fig 5:**
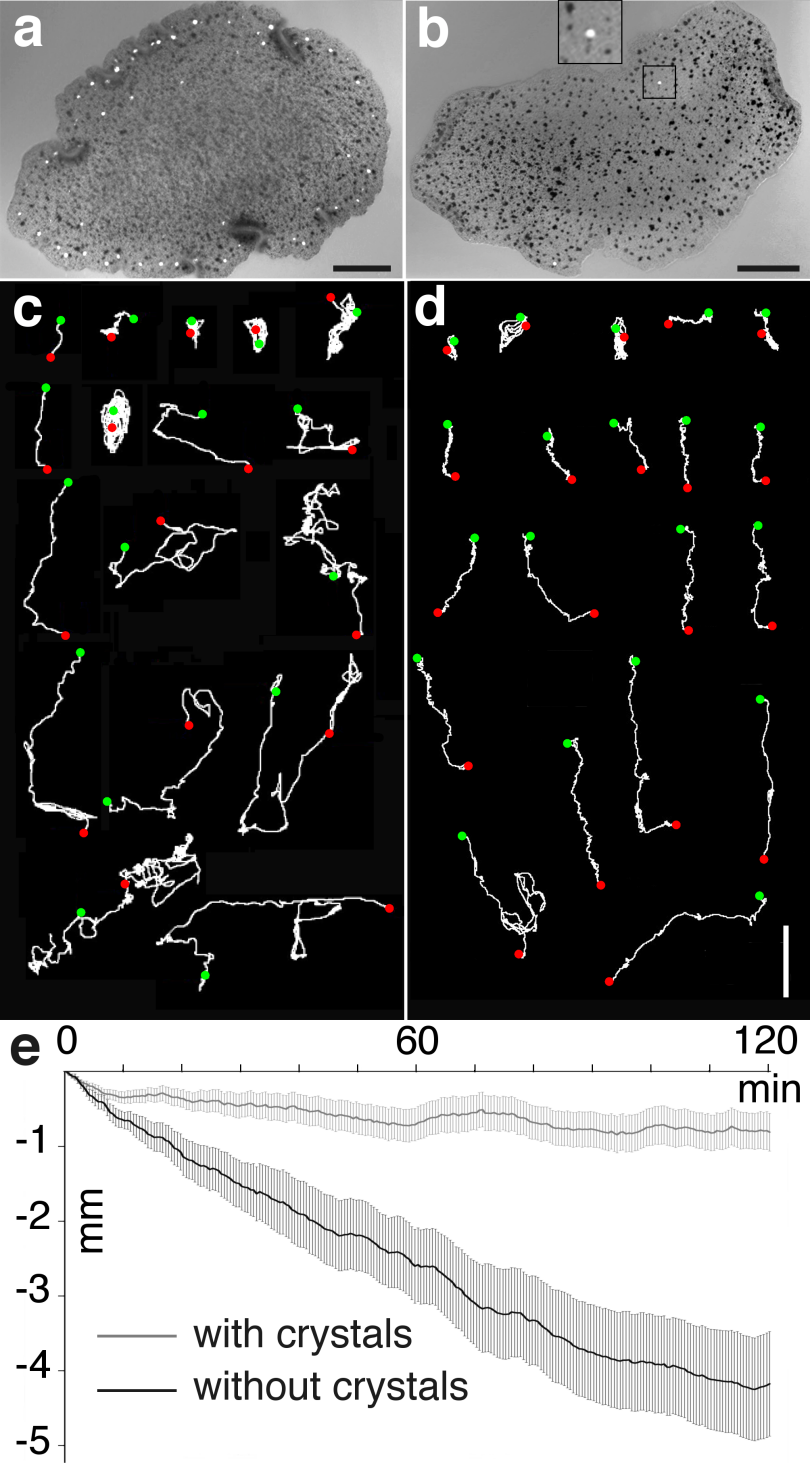
Abundance of crystal cells affects responses to changes in direction gravity. Transmitted polarized light image reveals numerous crystals arrayed around the edge of a typical animal (a), and only two crystals in an atypical animal (b). Inset (b) shows enlarged view of a crystal. (c), (d) Comparison of tracks of individual animals on a vertical substrate over the course of two hours, beginning 5 min after they were tilted vertically. Green and red dots mark, respectively, the beginning and end of each track. Tracks of many animals with a typical complement of crystals (c) include frequent episodes of movement in the direction opposing gravity (10 of 17 animals), while animals with <8 crystals (d) move predominantly in the direction of gravity (17 of 20 animals). (e) Comparison of net displacement over time on a vertical surface of animals containing a typical complement of crystals (light gray line; N = 49) and animals containing <8 crystals (dark gray line, N = 33). Animals with a typical complement of crystals on average show only a small downward displacement over the course of two hours whereas animals containing <8 crystals descend at a rate of ~2 mm per hour. Error bars are SEM. Scale bars: a, b– 100 μm; c, d– 5 mm.

The presence of an intercellular central stone that shifted with changes in the direction of gravity suggested that crystal cells could be gravity detectors. This was tested directly by tilting glass slides with attached animals up by 90°, and then recording their behavior for up to two hours with a macro camera. Animals were separated into two groups: those with a typical number of crystals (Fig 5A); and those with fewer than eight crystals (Fig 5B).

The behavior of typical animals on a vertical surface (Fig 5C) differed little from that on a horizontal surface (S3 Fig). Approximately two thirds of the animals migrated over distance of many millimeters (up to 4 cm) along paths that resembled the random walks typical of *Trichoplax* maintained in the absence of food [30]. The remaining third of the animals remained within a highly-circumscribed area (~1.7 mm) and spent much of the time rotating in place. Plots of the vertical net displacement typical of tilted animals showed that they moved down the vertical glass slide during the first 10–20 minutes after tilting, but then slowed their downward displacement (Fig 5E), so that their average net displacement was almost zero (Fig 5E). Thus, animals maintained their approximate vertical position during a second hour at about the same level (~0.7 mm ± 1.6 lower than the initial position; mean±SD, Fig 5E).

A minority of otherwise apparently normal *Trichoplax* manifested only a few or even no crystals (Fig 5B). We associate this phenomenon with a developmental stage in the renewal of the crystal cell pool. Normally, animals lacking crystal cells add new ones over several days (personal observation). These animals upon staining with Hoechst dye did not appear to have cup shaped nuclei (not shown) suggesting that the absence of crystals accompanies the absence of differentiated crystal cells. When *Trichoplax* with 0–8 crystals were tilted vertically, their movement differed markedly from that of animals with a normal complement of crystals. Over two thirds of the animals moved persistently downward, in the direction of gravity (Fig 5D). Moreover, their tracks were roughly linear unlike the meandering tracks of animals with a normal complement of crystals. The approximately 30% of animals that did not migrate downward underwent little displacement over the course of the experiment, spending much of the time rotating in place. Plotting the average of net displacement over time confirmed the persistent downward movement in uniform steps of animals with sparse crystals during the experiment (Fig 5E).

## Discussion

Gravity sensation in many metazoans as well as protists is mediated by cells that contain crystalline statoliths of varying compositions [11,17,18,20,24,26]. The presence of cells containing crystals in *Trichoplax* prompted the question of whether these *crystal cells* are equipped to sense gravity and if they are, whether the animals respond to changes in orientation relative to gravity. We applied a variety of light and electron microscopic techniques to clarify the detailed anatomy of the crystal cells, as well as their possible role in gravity sensation. Crystal cells, arrayed around the perimeter of the animal ~20 μm from the edge, are oriented such that the opening of their cup-shaped nucleus faces the outside of the animal. The nuclear envelop together with the interconnected endoplasmic reticulum cover about half of the internal surface of the plasma membrane on the side of the cell facing the interior. The crystal, located in the interior, is surrounded by 5–6 mitochondria. The cytoplasm surrounding the crystal is remarkably lucent and free of organelles. Analyses of the crystal composition and structure by electron microscopy, electron energy loss spectroscopy (EELS) and X-ray spectroscopy showed that the crystals are aragonite, and thus heavy enough to move in the direction of gravity. Indeed, comparison of the positions of crystals in cells of animals on a horizontal substrate with those on a vertical substrate showed that crystals move under the influence of gravity. The two types of cells that contact crystal cells, fiber cells and epithelial cells, could serve to anchor them and potentially receive signals transmitted from them. A small fraction of animals has very few crystal cells. Comparison of the behavior of such animals with animals having a typical complement of crystals provided evidence that crystal cells modulate the behavior of the animal in response to a change in orientation with respect to gravity. We provide evidence that *Trichoplax* senses and responds to gravity and that crystal cells are the gravity sensors.

### Role of crystal cells in sensing gravity

*Trichoplax* in our hands quickly sink to the bottom when placed in a dish containing seawater, indicating that they are denser than seawater. Nevertheless, animals on a vertically-tilted surface showed only a slight tendency to move downward, provided that they contained a typical number of crystal cells. They still migrated in all radial directions or rotated in place, similar to animals on a horizontal surface. In contrast, animals having few or no crystals continually glided downward, descending more than four mm over the course of two hours. That animals containing crystals resist sinking, while animals lacking the normal number of crystal cells sink, indicates that crystal cells have an essential role in maintaining the positioning of animals on tilted surfaces, likely by sensing the tilt and initiating compensatory movements.

### Comparison of crystal cells with lithocytes of other animals

*Trichoplax* crystal cells most closely resemble the lithocytes of some free-living flatworms including Catenulida, Nemertodermatida, Proseriata, and Lurus [20–23], and Acoela [24]. Crystal cells like the lithocytes in these worms are round, contain a hard crystal, and have an electron lucent cytoplasm with few organelles. The lithocyte in the Proseriata flatworms also has a very thin nucleus flattened against the plasma membrane [20]. Lithocytes in the lurid turbellarian worms, like those in *Trichoplax*, have a crystal surrounded with mitochondria [21]. Flatworm lithocytes are enclosed in parietal cells and it is thought the entire lithocyte moves under the influence of gravity. Neural processes penetrate the capsule surrounding the lithocyte and it is assumed that these neurons relay information about changes in position in response to gravity [20]. In contrast, *Trichoplax* crystal cells appear to be immobile and the crystal moves inside the crystal cell.

CaCO_3_ crystals ranging from 30–150 μm thick are found in some chitons [31,32], brittle stars [33] and extinct trilobites [34] where they are believed to focus light but the crystals in *Trichoplax* appear to be too small to function in this manner.

### Evolutionary and comparative aspects of crystal composition

X-ray and energy loss analysis of crystals revealed that *Trichoplax* crystals consist of calcium carbonate with no traces of magnesium, thereby conforming to the mineral form aragonite. Though calcite is more thermodynamically stable than aragonite, calcium carbonate is known to biomineralize as aragonite in seawater when the concentration of Mg relative to Ca exceeds a critical threshold [35–38]. As the Mg/Ca ratio in seawater changed across geologic eras, either calcite (in *calcite sea*) or aragonite (in *aragonite sea*) was favored for biomineralization [36–39]. The Placozoan ancestor of *Trichoplax* is considered to have diverged more than 600 [40], or even more than 720 million years ago [41,42], in the Ediacaran or Cryogenian periods, both characterized by aragonite seas [36,37]. The chemical composition of the sea started to change 542 million years ago, becoming a calcite sea by 525 million years ago [36]. Thus, the composition of modern *Trichoplax* crystals is consistent with the possibility that crystal cells appeared early during placozoan evolution.

However, cnidarians also are thought to have evolved 600–720 million years ago, contemporaneously with placozoans [41,42]. Calcium is present in the statoliths of many cnidarians, but it rarely binds carbonate anions, with medusae deploying calcium phosphate or calcium sulfate as a major component of their statoliths [43–46]. We find neither phosphorus nor even a trace of sulfur in the lithocytes in *Trichoplax*. Moreover, the overall organization of statocysts in Cnidarians [11,46], as well as in Ctenophores [47,48], has little in common with *Trichoplax* crystal cells. The statoliths of Acoela, whose lithocytes are more similar to crystal cells of *Trichoplax*, appear to be composed of calcium apatite [23]. On the other hand, statoliths of the vast majority of higher animals consist of CaCO_3_ in both its calcite (mammals) [49,50] and aragonite (mollusks, teleost fishes) [51–53] forms. It seems likely, given the morphological and compositional diversity of statocysts in different animal taxa, that gravity receptors independently evolved more than once, even in different species of flatworms [20].

### How crystal cells may function as gravity sensors

Crystal cells are generally oriented with their cup shaped nuclei pointing outwards the periphery of the animal. If this polarization is essential to their function, the crystal cells would be expected to be fixed in position by contacts with other cells, even though cells in the interior of the animal generally appear to be only loosely associated. Complete serial reconstructions show that crystal cells are embraced by fiber cells and their processes, as well at least one epithelial cell. The epithelial cells that contact crystal cells have a thicker layer of actin filaments around their circumference than adjacent epithelial cells. This thick actin cortex could fortify these cells and amplify mechanical coupling with adjacent epithelial cells [54].

**Fig 6:**
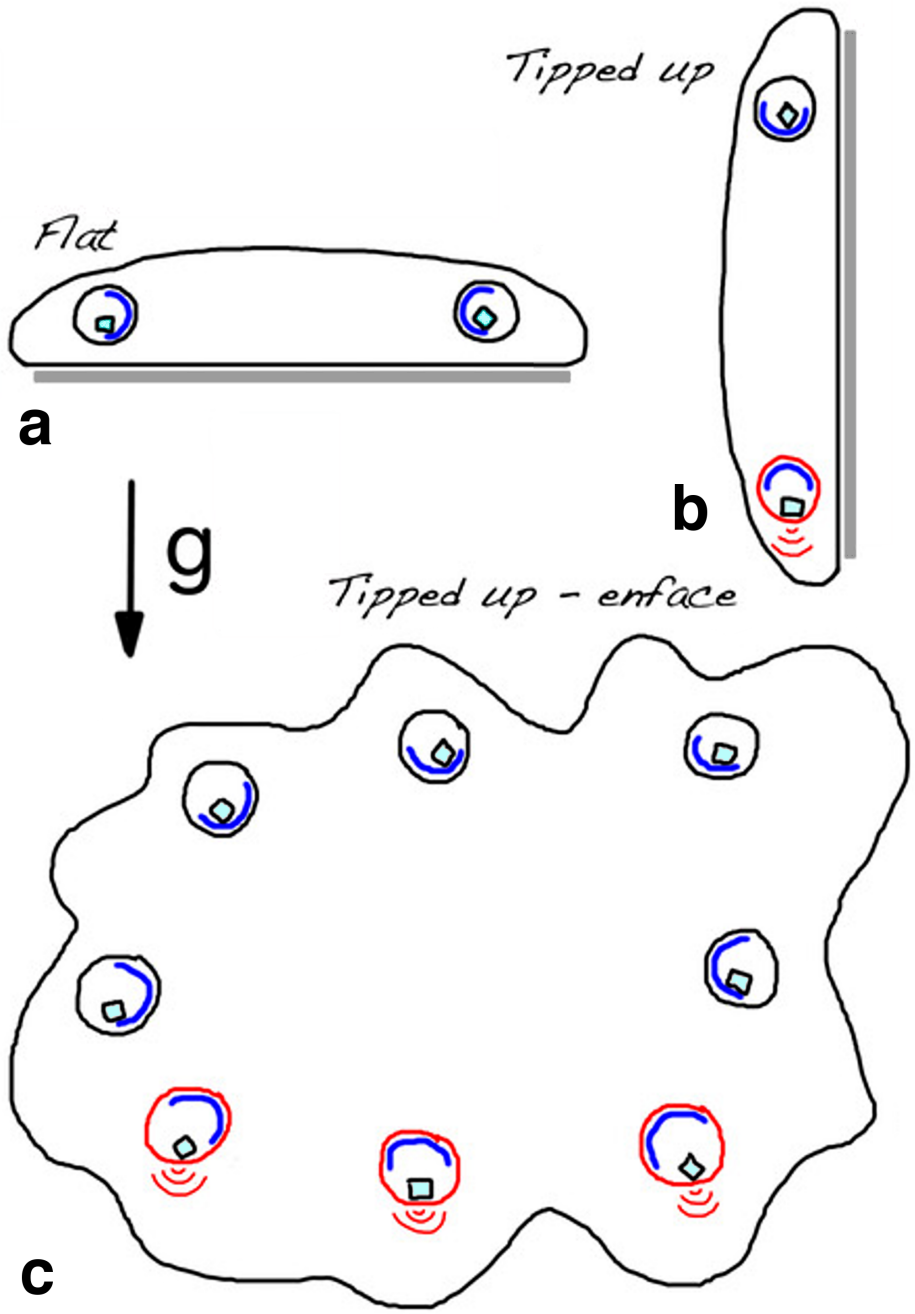
How crystal cells may be activated when tilted. Possible mechanism of selective crystal cell activation in Trichoplax upon being tilted vertically. Schematic cross-section of Trichoplax attached to horizontal (a) or vertical (b) substrates, and *en face* view of animal on a vertical substrate (c). Cup-shaped nuclei (blue) are directed to the edge of the animal, regardless of its orientation with respect to gravity, while crystals move under the influence of gravity. Crystals in animals on a horizontal surface (a) generally remain in the nuclear cup, but when the surface is tilted vertically (b), crystals on the down side of Trichoplax fall out of the cup (red outline). We postulate that pressure exerted by the crystal on the plasma membrane activates mechanosensitive receptors in the membrane, which in turn causes the crystal cell to transmit a signal (red brackets). *En face* view (c) illustrates the distribution of activated crystal cells (red outlines and brackets) in an animal on a vertical substrate.

Crystals inside crystal cells in animals on a vertical surface settle in the direction of gravity. Since the cup-shaped nuclei of crystal cells are oriented with their openings facing the edge of the animal, crystals in cells along the upper side of a tilted animal fall toward the nucleus while those in cells along the lower side move away from the nucleus and toward the plasma membrane on the opposite side of the cell (Fig 6). Mechanosensitive channels in their plasma membranes might be activated by direct contact with the crystal, or by the pressure of the crystal on the actin filaments lining the plasma membrane, which is known to facilitate mechanical impulses in plant statocytes [55]. The *Trichoplax* genome includes sequences homologous to mechanosensitive channels in bacteria [56] and animals [57]. In an animal on a vertical surface, the movement of the crystal in response to gravity would activate the mechanosensitive channels in crystal cells along the lower edge of the animal but not elsewhere (Fig 6). Alternatively, transduction could be initiated when the crystal settles into the nuclear cup and exerts pressure on the endoplasmic reticulum and nuclear envelop, as is thought to occur in statocytes of plant roots [58]. This hypothesis predicates activation of crystal cells along top of tilted animals.

It remains to be explained how a signal generated in a crystal cell could control the behavior of *Trichoplax*, which appears to have only a protonervous system [59]. Because there are a hundred or more crystal cells arranged around the periphery of the animal, control could be local, directly affecting only ciliated cells in the vicinity of each crystal cell. The prevalence of fiber cell processes in the vicinity of crystal cells might be indicative of a role for fiber cells in transmitting signals from crystal cells to the ciliated cells that power locomotion. However, the contacts of fiber cell processes with epithelial cells examined so far lack features suggestive of either electrical or chemical transmission [4,8].

Each crystal cell makes an extensive contact with a morphologically distinctive epithelial cell. Although these contacts also appear to lack apparatus for secretion, it remains possible that the crystal cell communicates directly with the epithelial cells by releasing ions [60] or small molecules [61] through channels directly into the narrow cleft between the two cells. The effect of the signal might be to alter the direction or frequency of beating of the cilia on the epithelial cell and, via mechanical or hydrostatic coupling, possibly also of cilia on adjacent cells.

In animals with multicellular statocysts innervated by multiple nerves (as in flatworms and Xenacoelomorpha [20,24,62]), signals coming from just one lithocyte, or a few lithocytes, can be transmitted and selectively distributed throughout the animal. We suggest that *Trichoplax* compensates for the absence of neural transmission by deploying a large number of crystal cells, each of which exerts local control over the beating of ciliated cells in its vicinity, most likely by direct communication with the epithelial cell that is in direct contact with each crystal cell.

Taken together, we present both structural and observational evidence that crystal cells mediate responses to changes of position of *Trichoplax* in relation to the direction of gravity. The anatomy of the crystal cells, the stable orientation of their nuclei, and a mobile crystal inside show how crystal cells might be selectively activated by tilting of the animal. Finally, comparison of the behavior of animals with decreased numbers of crystals, demonstrates that crystal cells influence responses to changes in the direction of gravity. The composition of the crystal (aragonite) is consistent with the possibility that crystal cells and gravity sensing may have evolved in the Cryogenian ancestors of placozoans.

## Supporting information

S1 Fig Thin section of crystal cell capturing fragments of crystal falling out of section. This fortuitous section shows that flat edges of the fractured crystal have separated cleanly from the interior of the crystal cell (arrow head). Mitochondria (asterisks) lie along the edge where fragments of the crystal have fallen out. This observation confirms that the crystal exists as a single entity in intact crystal cell [4]. SEM backscatter image from a whole animal prepared by freeze-substitution and sectioned with the ATUM. Scale bar– 1 μm.
(TIF)

S2 Fig Reconstruction from 123 serial sections of endoplasmic reticulum and other organelles in a crystal cell. Mitochondria (red) surround crystal (light blue). The crystal complex is found inside endoplasmic reticulum (yellow) cup, which constitutes the outer nuclear membrane. A Golgi apparatus (green) also lies in the endoplasmic reticulum cup. Light and dark vesicles depicted in light and dark pink. Crystal cell silhouette is shown in gray. This is the same crystal cell as rendered on Fig 1B, but from different point of view (rotated about 30° counter-clockwise around z-axis).
(TIFF)

S3 Fig Tracks of individual Trichoplax crawling on a horizontal glass substrate with no food. Green and red dots mark, respectively, the beginning and end of each track. Scale bar– 5 mm.
(TIF)

S1 Movie Movie of rotating 3D model of internal organization of the crystal cell. Blue–nucleus; light blue–crystal; red–mitochondria; green–Golgi complex; light and dark pink–electron light and dense vesicles, respectively.
(MP4)

S2 Movie Movie of rotating 3D model of internal organization of the crystal cell. Yellow–nuclear envelope giving rise to endoplasmic reticulum; light blue–crystal; red–mitochondria; green–Golgi complex; light and dark pink–electron light and dense vesicles, respectively.
(MP4)

S3 Movie Movie of rotating 3D model of external view of the crystal cell and adjacent cells. Note tight contact between crystal cell and processes of two fiber cells. Magenta–crystal cell; cyan–fiber cells (their fragments surrounding crystal cell); yellow–zones of tight contact between crystal and fiber cells; orange–epithelial cell (its basal part).
(MP4)

## Abbreviations

ASW: artificial sea water
ATUM: automated tape collecting ultramicrotome
EELS: electron energy loss spectroscopy
EELSI: electron energy loss spectroscopic imaging
ELNES: energy loss near-edge fine structure
EM: electron microscopy
HAADF: high-angle annular dark-field detector
PBS: phosphate buffered saline
PFA: paraformaldehyde
SEM: standard error of mean
SEM: scanning electron microscopy
STEM: scanning TEM mode
TEM: transmission electron microscope
