# Supplementary figures and images for: Cells containing aragonite crystals mediate responses to gravity in Trichoplax adhaerens (Placozoa), an animal lacking neurons and synapses
Source: PLoS One. 2018 Jan 17;13(1):e0190905. doi: 10.1371/journal.pone.0190905 (PMC5771587; doi:10.1371/journal.pone.0190905)

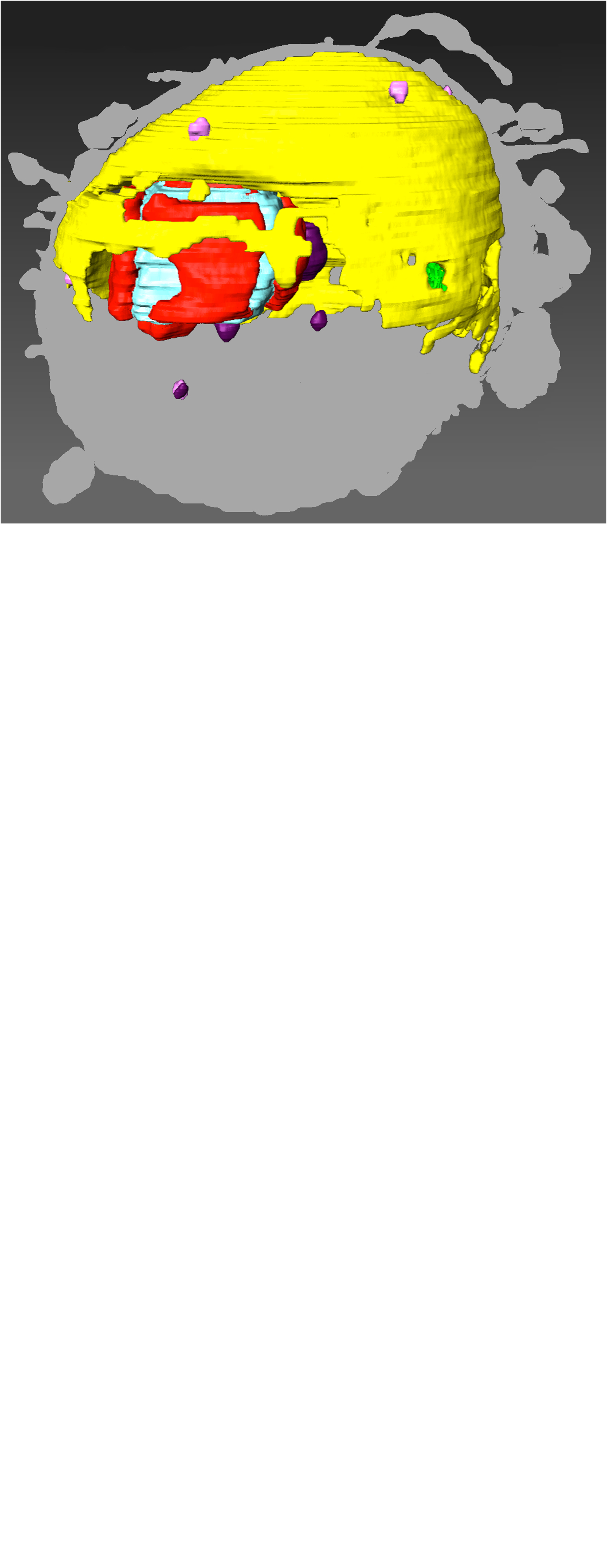

Supplement: S2 Fig — Mitochondria (red) surround crystal (light blue). The crystal complex is found inside endoplasmic reticulum (yellow) cup, which constitutes the outer nuclear membrane. A Golgi apparatus (green) also lies in the endoplasmic reticulum cup. Light and dark vesicles depicted in light and dark pink. Crystal cell silhouette is shown in gray. This is the same crystal cell as rendered on Fig 1B, but from different point of view (rotated about 30° counter-clockwise around z-axis). (TIFF) [file pone.0190905.s002.tiff]

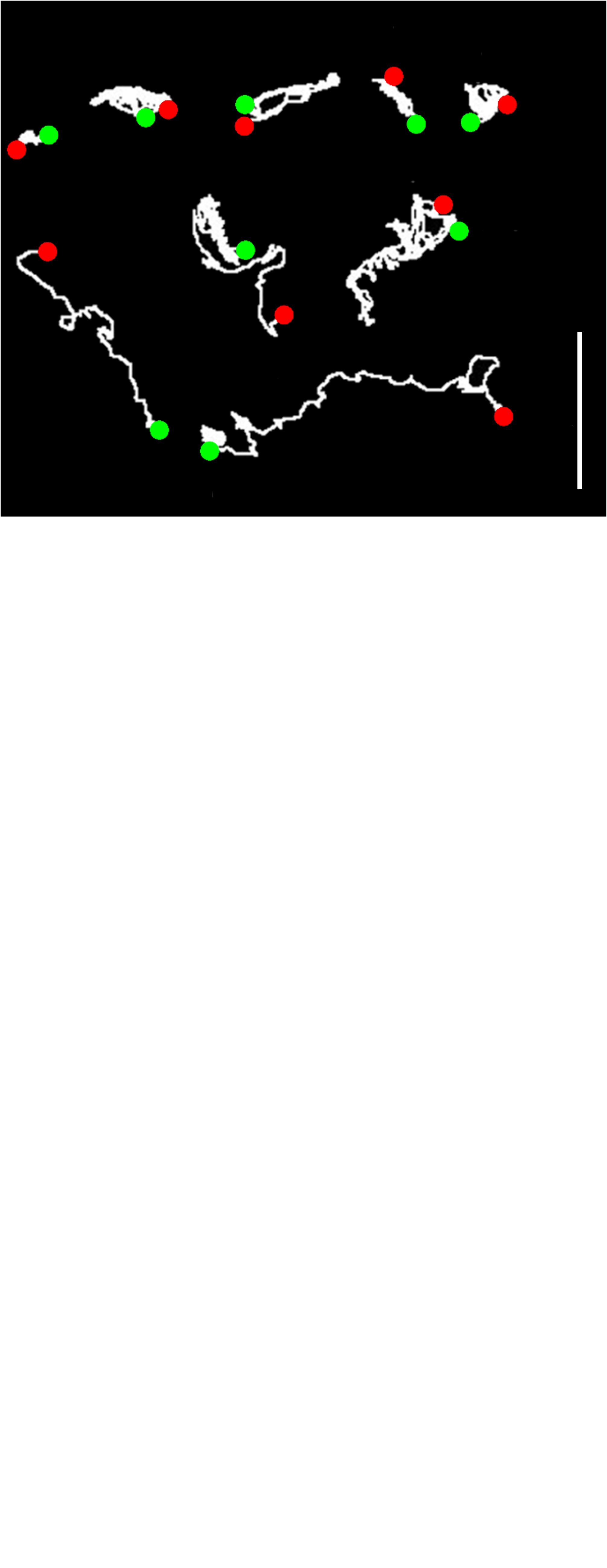

Supplement: S3 Fig — Green and red dots mark, respectively, the beginning and end of each track. Scale bar– 5 mm. (TIF) [file pone.0190905.s003.tif]
